# Patients over 40 years old with precursor T-cell lymphoblastic lymphoma have different prognostic factors comparing to the youngers

**DOI:** 10.1038/s41598-018-19565-x

**Published:** 2018-01-18

**Authors:** Meng Dong, Xudong Zhang, Zhenzhen Yang, Shaoxuan Wu, Mijing Ma, Zhaoming Li, Yu Chang, Xinhua Wang, Ling Li, Xin Li, Mingzhi Zhang, Qingjiang Chen

**Affiliations:** Department of Oncology, The First Affiliated Hospital of Zhengzhou University, Zhengzhou, 450052 PR China

## Abstract

This study aimed to analyze the clinical characteristics and prognostic factors of patients, divided into over 40-year-old group or not, with precursor T-cell lymphoblastic lymphoma (Pre-T-LBL). Based on the retrospective analysis of the clinical data of 59 patients with Pre-T-LBL during the period from December 2010 to December 2015, albumin level, anemia, pleural or pericardial effusion, protocol, therapy response, mediastinal mass, lactate dehydrogenase (LDH), and international prognostic index (IPI) or age-adjusted international prognostic index (aaIPI) were summarized. For patients aged <40 years, factors correlating with poor progression-free survival (PFS) were pleural or pericardial effusion, regimen, albumin level and therapy response. Pleural or pericardial effusion, aaIPI score, regimen, LDH increased, albumin level, therapy response and mediastinal mass were all related with poor overall survival (OS). In the patients aged ≥40 years, only anemia associated with PFS. However, anemia, involvement of bone marrow and therapeutic response were all related with poor OS. In conclusion, the patients with Pre-T-LBL are characterized by a low incidence and bad prognosis. Different prognostic factors can be discovered for patients over 40-year-old with Pre-T-LBL comparing to the youngers. New prognostic evaluation factors should be explored for patients ≥40 years old.

## Introduction

Precursor T-lymphoblastic lymphoma/leukemia (Pre-T-LBL) comprises approximately 85%-90% of all lymphoblastic lymphoma (LBL)^1^. According to 2016 World Health Organization (WHO) classification of hematopoietic and lymphoid tumors^2^, precursor acute lymphoblastic leukemia (Pre-ALL) and LBL currently are considered as two different manifestations of the same disease. It frequently presents a typical mediastinal mass, fever and lymphadenectasis at diagnosis, with or without superior vena cava syndrome, which has an aggressive clinical course and worse prognosis than other dieases^3^. Huguet *et al*.^4^ reported pediatric-inspired therapy improved the outcome of adolescent patients with Pre-ALL, while the upper age was unclear. Ellin *et al*.^5^ described that age was one of the significant adverse factor for overall survival (OS) and progression free survival (PFS). And Zhu *et al*.^6^ demonstrated that satisfactory OS cann’t be received for patients less than 40 years old. Surprisingly, there has been no comparison for different age groups with Pre-T-LBL. Herein, we analyzed the clinical data of 59 patients from our hospital to evaluate the clinical characteristics and to reveal prognosis factors for patients greater than or equal 40 years old and less than 40 years old.

## Results

### Patient characteristics

**Figure 1:**
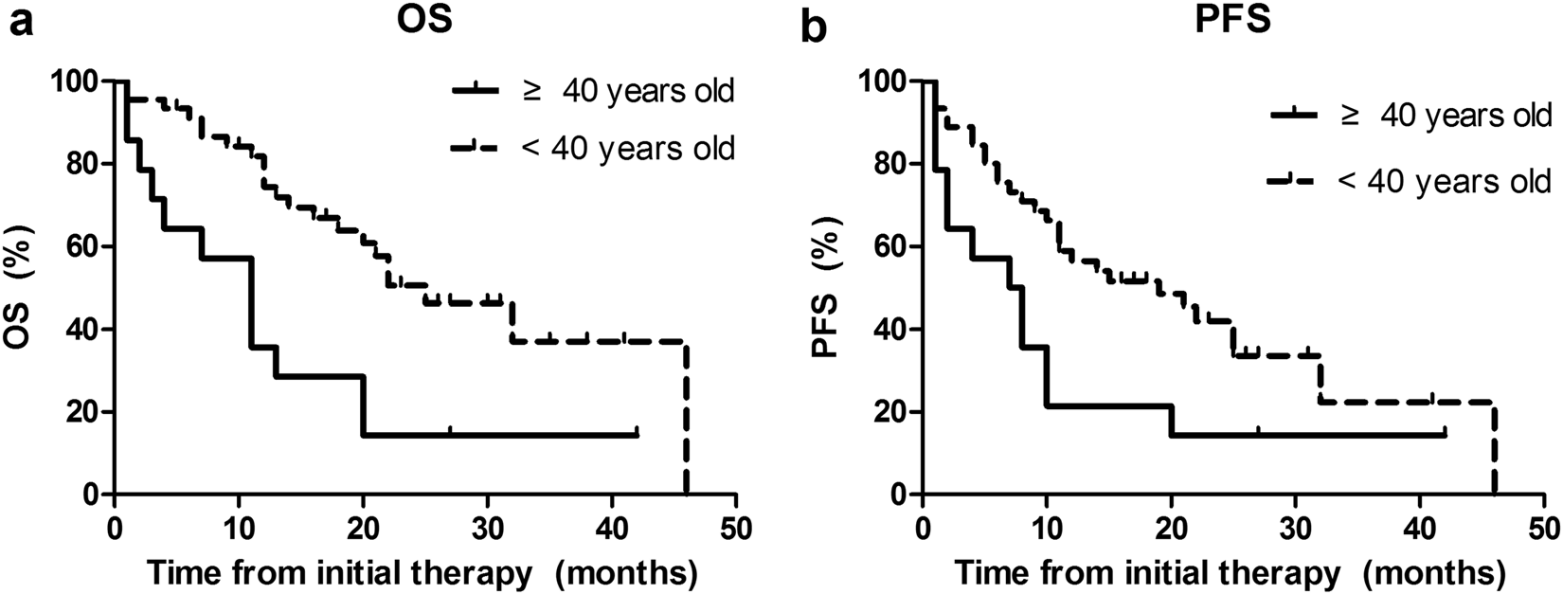
Kaplan-Meier curves for analysis of OS (**a**) and PFS (**b**) among 59 patients with Pre -T-LBL.

The main clinical characteristics of the 59 patients were presented in Table 1. The median age was 27 years old (range: 5–77 years old) with 76.3% under 40 years old. The distinct difference can be observed in Fig. 1 (OS *P* = 0.002 and PFS *P* = 0.024, respectively). The male: female ratio was 4.9:1. Thirty-nine patients (66%) had a good Eastern Cooperative Oncology Group (ECOG) score of 0–2. Using the Ann Arbor staging system, 49 patients (83%) presented with stage III or IV disease. B symptoms were reported in 24 patients (41%) at diagnosis. Fifty-three patients (90%) had a mediastinal mass. BM involvement was common, occurring in 46% of all patients. LDH level was frequently elevated. Based on IPI scores, less than half of the patients had low or low-to-moderate risk disease. Intrathoracic effusions were most frequently observed in 60% of patients, particularly. Conversely, only 29% of patients greater than or equal 40 years old had intrathoracic effusions (*P* = 0.04). With regard to stage, age, BM involvement, ECOG score, B symptoms and Ann Arbor stage, no significant difference was observed between different age groups patients with Pre-T-LBL.

**Table 1:** Clinical data in 59 T-cell Lymphoblastic Lymphoma patients.

	All Pre-T-LBL patients(n = 59)	<40 years Pre-T-LBL patients(n = 45)	>=40 years Pre-T-LBL patients(n = 14)	*P* value
**Sex**				1.000
Male	83.1% (49/59)	82.2% (37/45)	85.7% (12/14)	
Female	16.9% (10/59)	17.8% (8/45)	14.3% (2/14)	
**Stage**				0.917
1–2	16.9% (10/59)	15.6% (7/45)	21.4% (3/14)	
3–4	83.1% (49/59)	84.4% (38/45)	78.6% (11/14)	
**LDH increased**				0.774
Y	61.0%(36/59)	60.0% (27/45)	64.3% (9/14)	
N	39.0%(23/59)	40.0%(18/45)	35.7% (5/14)	
**Intrathoracic effusions**				0.040
Y	52.5% (31/59)	60.0% (27/45)	28.6% (4/14)	
N	47.5% (28/59)	40.0% (18/45)	71.4% (10/14)	
**Bone marrow**				0.803
Y	45.8% (27/59)	46.7% (21/45)	42.9% (6/14)	
N	54.2% (32/59)	53.3% (24/45)	57.1% (8/14)	
**ECOG**				0.626
0–2	66.1% (39/59)	68.9% (31/45)	57.1% (8/14)	
3–5	33.9% (20/59)	31.1% (14/45)	42.9% (6/14)	
**B symptoms**				0.416
Y	40.7% (24/59)	37.8% (17/45)	50.0% (7/14)	
N	59.3% (35/59)	62.2% (28/45)	50.0% (7/14)	
**Therapy**				0.027
Hyper CVAD	6.4% (31/55)	50.0% (22/44)	81.8% (9/11)	
DA-BFM 90	52.7% (29/55)	61.4% (27/44)	18.2% (2/11)	
**Therapy Response**				0.698
CR	56.1% (23/41)	57.6% (19/33)	50.0% (4/8)	
NCR	43.9% (18/41)	42.4% (14/33)	50.0% (4/8)	

Abbreviations: LDH, lactate dehydrogenase; CHOP or CHOP like, cyclophosphamide, adriamycin, vincristine and prednisone; ALL or ALL like: BFM-90 or Hyper-CVAD; CR: complete remission; NCR: not complete remission.

### Treatment response

**Figure 2:**
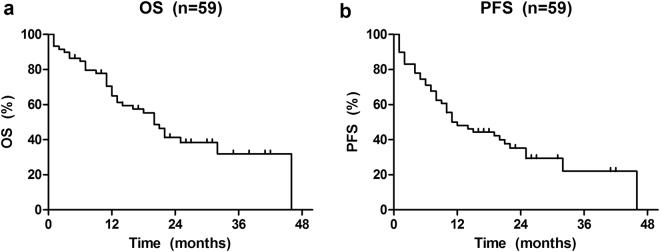
Kaplan-Meier curves for analysis of OS (**a**) and PFS (**b**) of age among 59 patients with Pre -T-LBL.

Within a median follow-up time of 16 months (range: 1–46 months), the 3-year OS and PFS rate for all patients were 31.9% and 22.0%, respectively (Fig. 2a and b).

Four cases (6.8%) had no treatment in our hospital. Six patients (10.9%) had radiotherapy followed chemotherapy. In the subgroup with less than 40 years old, majority of patients received ALL regimens including DA-BFM 90 (52.7%) and Hyper CVAD (56.4%), however, one patient wasn’t treated. On the other hand, 78.6 percentage (n = 11) of patients received ALL protocol. There also were 3 patients having no treatment. CR was observed in 56.1% (n = 23) whereas 43.9% (n = 18) of cases displayed PR, persistent disease and died early. There were no significant differences in the frequency of these characteristics between the two age groups.

### Prognostic factors

Among the patients less than 40 years old, the possible predictive factors of survival were summarized in Table 2. Factors correlating with poor PFS were intrathoracic effusion (*P* = 0.005), regimen (*P* = 0.023), albumin level (*P* = 0.003), therapy response (*P* = 0.003). It was found that intrathoracic effusion (*P* = 0.006), high IPI or aaIPI score (*P* = 0.012), regimen (DA-BFM 90 or Hyper CVAD, *P* = 0.044), LDH level (*P* = 0.006), albumin at diagnosis (*P* = 0.019), therapy response (*P* = 0.005) and mediastinal mass (*P* = 0.038) were all related with poor OS. Although IPI or aaIPI score and LDH level at diagnosis were associated with OS, a gradual rise in LDH level and IPI or aalPI score were really unassociated with the PFS (Table 2). Whereas, in the patients no less than 40 years old, there were prognosis factors including anemia at diagnosis (*P* = 0.003), involvement of bone marrow (*P* = 0.020) and therapeutic response (*P* = 0.029) which were all related with shorter OS. Prognosis factors of OS weren’t associated with PFS except anemia at diagnosis (*P* = 0.017, Table 2). However, in the less than 40 years old group, TdT positive expression (*P = *0.314) and Ki-67 ≥ 80% (*P* = 0.232) had no influence on PFS. They also had no obvious correlation to OS (*P = *0.973, *P = *0.628). The same results could be got in the greater than or equal to the age group of 40 years group. Which is that TdT (*P* = 0.835) and Ki-67 ≥ 80% (*P = *0.617) had no influence on PFS. In term of OS, the same result could be obtained (*P = *0.936, *P = *0.268, Table 2).

**Table 2:** Univariate analysis of survival in patients with Pre-T-LBL.

Paramater	<40 years old		>=40 years old	
	OS *P* value	PFS *P* value	OS *P* value	PFS *P* value
Intrathoracic effusion	0.006	0.005	0.758	0.155
IPI or aaIPI	0.012	0.114	0.600	0.735
LDH level	0.006	0.163	0.829	0.774
Albumin level	0.019	0.003	0.705	0.157
Therapy Response	0.005	0.003	0.029	0.069
Mediastinal mass	0.038	0.086	0.996	0.619
Anemia level	0.361	0.433	0.003	0.017
Bone marrow involvement	0.866	0.796	0.020	0.109
Regimen	0.044	0.023	0.585	0.544
TdT positive	0.974	0.312	0.936	0.312
Ki-67 ≥ 80%	0.628	0.232	0.268	0.617

The step back Cox proportional hazard model was used to determine the independent prognostic risk factors for patients less than 40 years old. Therapy response and albumin level were important independent OS predictors. Independent prognostic factor for PFS is therapy response (Table 3). For the patients no less than 40 years old, independent predictors of PFS and OS couldn’t obtain because the number of samples wasn’t enough for Cox proportional hazard model.

**Table 3:** Multivariate analysis of survival in patients with Pre-T-LBL.

Paramater	<40 years old		>=40 years old	
	RR (95% CI)	*P* value	RR (95% CI)	*P* value
OS				
Therapy Response	4.431 (1.040–13.881)	0.044	NA	NA
Albumin level	0.171 (0.032–0.921)	0.04	NA	NA
PFS				
Therapy Response	5.229 (1.48–18.473)	0.043	NA	NA

## Discussion

LBL is a rare subtype of non-Hodgkin lymphoma (NHL) comprising about 2–4% of NHL cases in adults. Approximately 85–90% of LBL in adults is of the T-cell phenotype^7^. In children, Pre-T-LBL accounts for about 1/3 of pediatric NHL^8^. Our report described the results achieved among 59 Pre-T-LBL patients including no less than 40 years old and the younger. The reported median age at diagnosis in adults was between 20 and 40 years old, with male predominance^7,9^. In our study, the median age at diagnosis was 27 years old, with male predominance, too (M: F = 4.9:1). Typical clinical features of Pre-T-LBL contained bulky mediastinal disease, with a propensity for the involvement of BM and central nervous system (CNS), and pleural effusions. The clinical characteristics of the two groups patients with Pre-T-LBL analyzed in present study were similar to those previously described apart from intrathoracic effusions. It had a significantly different between patients less than 40 years old and more than 40 years old (*P* = 0.040). The stronger immunity of adolescent than elderly should be explained these differences. Stage was often advanced at diagnosis (stage III-IV)^10^, which are coincident with those observed in our series.

Prognostic markers in Pre-LBL are yet to be clearly defined. Age, gender, response to treatment, stage, molecular prognostic markers, BM and CNS involvement remain unclear as prognostic factors in Pre-T-LBL. The outcome was inferior for pleural or pericardial effusion positive patients comparing with negative ones in adolescent. And in a recent study by Burkhardt^11^, adolescent females with Pre-T-LBL had a worse outcome.

As for IPI or aaIPI score, patients younger than 40 years old with high scores had a poor prognosis. Low IPI or aaIPI score didn’t company with better PFS in both subgroups (*P* = 0.114 and 0.734, respectively). However, the score was a prognostic factor meaning significantly better OS observed in patients less than 40 years old, as compared to those more than 40 years old. The outcome was similar to the those of the study operated by Yang^12^. The level of LDH was also an important prognostic factor for children and adolescent patients. In term of overall survival rate, increasing of LDH mean poor outcome, which had no enough significance for elderly patients. However, there was no evidently different between two groups for PFS. Statistical analysis indicated that mediastinal mass was concerned with poor OS and unconnected with PFS for young adults and children, which was analogous to the consequence of Jin^13^. Albumin level was also much more important for patient at the age <40 years either OS or PFS. On the contrary, we couldn’t see the result in elderly patients because of the lower morbidity. Response to treatment was considered an important prognostic factor. In a series of 121 children with Pre-T-LBL who had reached CR on the seventh day of chemotherapy had a better prognosis than those who had not reached CR^14^. Our present study showed that patients receiving CR had much significantly better than no complete remission (NCR) after induction therapy for both subgroups.

Anemia and involvement of BM were poor prognostic factors for elderly patients. Nevertheless, those had no relationship with outcome of patients less than 40 years old. The findings can be compared with that reported previously^6,13^. Majority patients received ALL-like regimen such as DA-BFM-90 and Hyper-CVAD. The CR rate was 56.1% in all patients. Meanwhile, the rate was similar to each other for two subgroups (57.6% and 50.0%, respectively). Recently, Zhu^6^ demonstrated that childhood chemotherapy regimen improved the overall survival rate more than the adult regimen in patients aged <40 years. In our cohort, the result was coincident with those observed previously. Instead, in patients ≥40 years old, childhood chemotherapy regimen (DA-BFM 90) had no difference with adult chemotherapy protocol (Hyper CVAD). Hematopoietic stem cell transplantation (HSCT) played a key role in patients with high-risk or relapsed/refractory disease. Yang^12^ suggested that chemotherapy combined with HSCT give a much better than chemotherapy alone. In our study, all patients getting HSCT were adolescent and young adult (AYA) suggesting no advantage. With the success of the intensification of the therapy in AYA patients with ALL, the outcomes of Ph-ALL patients now superior than that of allogeneic hematopoietic stem cell transplant (Allo-HSCT) in first CR^15^. For elderly patients, HSCT remain an important component of the treatment of precursor T-cell lymphoblastic leukemia (Pre-T-ALL) for patients with high-risk or relapsed/refractory disease^16^.

Several works shown superior outcomes for adolescent and young adult patients with ALL when treated with pediatric regimens, as compared with adult regimens^17–19^. The Meta-analysis of Kako^20^ demonstrated that the use of Dexamethasone (Dex) and high-dose L-asparaginase (L-asp) or methotrexate (MTX) may improve the outcome of T-lineage ALL.

With the development of medical technology, several novel strategies were found for Pre- T-LBL/ALL. The work of Richard^21^ suggested that combined targeting of tyrosine kinases and activation of serine/threonine phosphatases may offer novel therapeutic strategies for the treatment of Pre-T-ALL. Moreover, antibody of PD-L1 may improve the outcome of Pre-T-LBL/ALL according to the study of Miyoshi^22^. In addition, active mutation of NOTCH1, JAK2, PTEN and LOH6q were reported to be associated with favorable prognosis^23–25^. In our study, TdT positive expression and Ki-67 ≥ 80% had no influence on PFS and OS. The results also could be found either in patients less than 40 years old group or no less than 40 years old. Thus, it was difficult to find an effective relationship with the prognosis by conventional immunohistochemistry. All in all, further research about cytogenetics is urgent need to find related molecules related with prognosis.

According to the 2016 revision of the WHO classification of lymphoid neoplasm, although they have different clinical presentations, Pre-T-ALL and Pre-T-LBL are considered to be the same disease^2^. However, Pre-T-ALL mainly violates blood and bone marrow. And much more tumor cells can be definite in bone marrow (≥20%). T-LBL cells can be found in lymph nodes and mediastinal mass, tumor cells are rarely found in bone marrow^16^. However, the method is so simple that researchers have to pay more attention to relationship between molecular mechanism and prognosis.

PTEN and LOH6q mutation is poor prognosis factor of T-LBL respectively. The mutation of NOTCH1 is good for the patients. Both PTEN and LOH6q mutation lead to poor prognosis. What’s more, both mutation of PTEN and NOTCH1 at the same time also result in much poorer prognosis^25^. The researches had been reported about the relationship between JAK family mutation and Pre-T-ALL/LBL. JAK1, JAK2 affected the occurrence of Pre-T-ALL/LBL by activating IL7R/JAK-STAT signaling pathway^16^. JAK2 mutation in H574R and R683G and methylation of SOCS3 were able to promote cell proliferation in the way of activating the JAK-STAT pathway. Apart from this, T cell receptor (TAL1, LMO1, LMO2, TLX1, TLX3) translocation mutations led to abnormal expression of transcription factors. The deletion mutation of transcription factors (WT1, LEF1, RUNX1, ETV6), epigenetic tumor suppressor (EZH2, SUZ12, PHF6) and cell cycle inhibitory factors (CDKN2A, RB, CDKN1B) can result in T-LBL/ALL^16^.

Although Pre-T-ALL and Pre-T-LBL are currently considered to be the same disease with different clinical manifestations, different clinical manifestations maybe indicate different genetic factors. The reason for different clinical manifestations was differences in chemotaxis and vascular regeneration according to the study of Katia^26^. The deregulation of ARRB2 is related to chemotaxis of Pre-T-LBL. The mutation of SLIT2 and the upregulation of EPAS1 and PTPRB, which can promote blood vessel regeneration in Pre-T-LBL, didn’t be found in Pre-T-ALL. The expression of MLL-1 in Pre-T-LBL was much higher than that in Pre-T-ALL. On the contrary, the expression of CD47 in Pre-T-ALL was much higher than that in Pre-T-LBL^27^.

There are still some circumscribed elements in our research. Firstly, the number of samples was limited, expecially for the patients no less than 40 years old. Secondly, it was vital to pay attention to gene for Pre-T-LBL and more work need to be taken in oder to further outcomes.

In conclusion, our findings indicated that intrathoracic effusion, IPI or aaIPI, regimen, the level of LDH, albumin level, bulky mass and therapy response were prognosis factors of Pre-T-LBL. For patients less than 40 years old, therapy response and albumin level were independent prognosis factors. As for patients aged ≥40 years old, prognosis factors included anemia at diagnosis, involvement of bone marrow and therapeutic response. New prognostic evaluation factors should be explored for patients aged ≥40 years.

## Materials and Methods

### Patients

Fifty-nine patients with precursor T-lymphoblastic lymphoma/leukemia (Pre-T-LBL) were diagnosed at the First Affiliated Hospital of Zhengzhou University from 12/2010-12/2015. All patients were divided into two groups (≥40 vs <40 year old) according to older or younger than 40 years. All biopsy specimens were reviewed and confirmed according to the WHO criteria^16^ for Pre-T-LBL diagnosis. The pathological sections were reclassified by hematopathologist. The criteria for case inclusion were: (1) Histologically confirmed diagnosis of Pre-T-LBL (Cells with diffuse growth and medium size, have a small amount of cytoplasm. The entire structure of the lymph nodes is destroyed and accompanied by the film involved. And “Star” phenomenon can be found. In the part of the subcortical area involving, residual germinal center can be seen. Distorted nuclei, mitotic figures can be discovered in most of the primitive cells. (2) Pre-T-LBL cell type confirmed using immunohistochemistry or flow cytometry (Pre-TdT positive with variable expression of CD1a, CD2, CD3, CD4, CD5, CD7, and CD8. Cytoplasmic CD3 and CD7 are often positive). (3) No previous malignancy. (4) No previous treatment for lymphoma. (5) Adequate clinical information.

On paraffin sections, the positivity of terminal deoxynucleotidy transferase (TdT) had been observed in 86.5% of cases. In addition to TdT, all cases variably expressed CD3, CD4, CD5, CD7, CD8, CD34 and CD99. It’s necessary for TdT negative expression cases to detect other markers, such as CD99, CD34. Before therapy, the following clinical procedures should be performed: physical examination, laboratorial tests including complete blood count, serum lactate dehydrogenase (LDH), bone marrow (BM) examinations, computerized tomography (CT) or positron emission computed tomography (PET) of the chest, abdomen, pelvis. What’s more, the international prognostic index (IPI) or age adjusted international prognostic index (aalPI) were also evaluated. The clinical staging was defined in accordance with the Ann Arbor system. Both the institutional review board and ethics committees of First Affiliated Hospital of Zhengzhou University approved this study.

### Treatment and response assessment

Almost all patients received acute lymphoblastic leukemia (ALL) regimens, including dose-adjusted BFM-90 (DA-BFM-90) which was derived from BFM-90 (prednisone, Vincristine, daunorubicin, L-Asaraginase, cyclophosphamide, cytarabine, bleomycin), Hyper-CVAD (cyclophosphamide, vincristine, doxorubicin, and dexamethasone, alternating with high-dose methotrexate, and cytarabine). All the patients exhibited III/IV grade myelosuppression during remission induction. No death occurred due to toxicity. The treatment response was assessed after induction protocols of DA-BFM-90 or Hyper CVAD. According to Cheson 1999 criteria^28^, the curative effect was evaluated for complete remission (CR), partial remission (PR) and no response (NR).

### Statistical analysis

Overall survival (OS) was calculated from the time of diagnosis until death from any cause. Progression free survival (PFS) was defined as the interval from the initiation of chemotherapy to the time of the first documented disease progression or recurrence. All patients underwent complete and effective follow-up. The Chi-square test was used to calculate statistical group comparisons of categorical variables. Survival analysis was performed using the *Kaplan-Meier* method, with comparisons using the *log-rank test*. Multivariate analysis with a *Cox regression model* was used to estimate the prognostic impact of different variables on OS and PFS. Value of *P* < 0.05 was considered significant, and all *P* values corresponded to two-sided tests. All statistical analyses were performed using SPSS 17.0 software.
